# Long-term refractive outcomes of posterior chamber phakic (spheric and toric implantable collamer lens) intraocular lens implantation

**DOI:** 10.1007/s10792-013-9860-1

**Published:** 2013-10-11

**Authors:** Arturo Gomez-Bastar, Martha Jaimes, Enrique O. Graue-Hernández, Tito Ramirez-Luquin, Arturo Ramirez-Miranda, Alejandro Navas

**Affiliations:** Department of Cornea and Refractive Surgery, Institute of Ophthalmology “Conde de Valenciana”, Chimalpopoca 14, Col Obrera, 06800 Mexico City, Mexico

**Keywords:** Posterior chamber intraocular lenses, Implantable collamer lens, Phakic IOLs, ICL, TICL, pIOLs

## Abstract

To report the long-term refractive outcomes, safety, predictability, efficacy and complications of 349 eyes treated with posterior chamber phakic intraocular lenses (pIOLs). A retrospective review of consecutive clinical cases of patients who underwent spheric implantable collamer lens (ICL) and toric ICL (TICL) implantation. The study included 349 eyes of 216 patients with sphere between +8 to −24 diopters (D) and 0 to −6.5 D of astigmatism. Statistical analysis was performed to identify differences between preoperative and postoperative refractive outcomes. Main outcome measures were preoperative and postoperative uncorrected distance visual acuity (UDVA), corrected distance visual acuity (CDVA), spherical and cylindrical errors and spherical equivalent and significant postoperative complications. 194 eyes were treated with TICL and 155 eyes with ICL. The mean age of the patients was 29 ± 6.7 years. The mean preoperative sphere was −10.35 ± 5.1 D (+8 to −24) and the postoperative sphere was −0.09 ± 1.06 D (+3.25 to −6.5), *p* < 0.001. Preoperative cylinder was −2.63 ± 1.44 (0 to −6.5 D) and postoperative cylinder was −0.97 ± 0.89 D (0 to −3.5), *p* < 0.001. The preoperative mean spherical equivalent was −11.6 ± 5.12 D (+7.875 to −25.625) and postoperative spherical equivalent was −0.52 ± 1.03 (+2.25 to −6.75), *p* < 0.001. The mean preoperative UDVA was 1.72 ± 0.49 and postoperative UDVA was 0.23 ± 0.22, *p* < 0.001. The mean preoperative CDVA was 0.21 ± 0.17 and postoperative CDVA was 0.12 ± 0.138, *p* < 0.001. The implantation of posterior chamber pIOLs is a safe, predictable and effective strategy to manage refractive errors during long-term follow-up.

## Introduction

Several severe complications have presented with phakic intraocular lens (pIOL) implantation in both anterior chamber and posterior chamber models, including pupil ovalization [1], corneal decompensation [2, 3], glaucoma, cataract formation [4, 5], dislocation to the vitreous cavity [6] and retinal detachment [7].

However, a sudden increase in the popularity of pIOLs has occurred in the last several years, after studies by the United States Food and Drug Administration (FDA). To date, they have approved only two pIOLs—Verisyse (Advanced Medical Optics, Inc., Santa Ana, CA, USA) [8] in 2004 and Visian ICL^®^ (STAAR Surgical, Monrovia, CA, USA) [9] in 2008. Since then, newer models and materials have been developed and designed including Acrysof Cachet (Alcon Laboratories, Inc, Ft Worth, TX, USA) [10] and Epi.Lens (Acri.Tec/Carl Zeiss, Meditec, Jena, Germany) [11] due to the potential benefits of pIOLs.

Phakic visian toric implantable collamer lens (STAAR Surgical) is a foldable collamer lens designed to correct myopia and astigmatism and is under clinical trials for FDA approval [12]. International available power varies from −3 to −23 for sphere and from +1 to +6 for cylinder.

While an understandable caution exists regarding pIOL implantation, unfortunately risks also remain. We present a retrospective review of a consecutive clinical case series study of 349 eyes of 216 patients who underwent implantable collamer lens (ICL) or toric implantable collamer lens (TICL) implantation with overall favorable outcomes.

## Materials and methods

A retrospective chart review of a consecutive clinical case series study performed at the Instituto de Oftalmología ‘Fundación Conde de Valenciana’, Mexico City, Mexico consisting of 216 patients (349 eyes) with the diagnosis of myopia, hyperopia, or myopic or hyperopic astigmatism treated with Visian ICL^®^ between 2000 and 2011. Procedures were approved by the Institution’s Ethics Committee. Before 2005, all patients were treated with spheric models; however, after 2005, patients received toric and spheric models as needed for correction of astigmatic errors (>2 D of cylinder to consider TICL model). Patients with at least 1 month of follow-up were included in the data analysis. The initial selection criteria included patients whose laser vision correction (LVC) was contraindicated due to small residual bed, high-calculated ablation and/or abnormal topographies. The subjects were required to be at least 18 years old with a stable refraction and without any evidence of ophthalmological diseases. All patients underwent refraction, complete ophthalmological evaluation including but not limited to intraocular pressure, gonioscopy, fundoscopy, pachymetry, endothelial cell count, and pupillary diameter. Topographic values were obtained with Orbscan II (Orbtek, Bausch & Lomb, Rochester, NY, USA) and endothelial cell count with specular microscopy (CellCheck XL™, Konan Medical, Hyogo, Japan). A minimum endothelial cell count of 2,000 cells/mm^2^ and anterior chamber depth (ACD) of at least 2.8 mm (by means of Orbscan II) were included.

Biometric measurements have a key role in ICL calculations and final ICL (vault) position. We routinely used white-to-white (W–W) distance, ACD and keratometric values obtained by Orbscan II (this may vary with different ethnic and race groups). The most important exclusion criteria was ACD <2.8 mm (measured from the endothelium: Orbscan II can be adjusted to display both epithelial or endothelial ACD).

For ICL and TICL software calculations we used manifest refraction in all cases. For vertex distance we selected 12 mm for all cases. Orbscan II values were used for keratometries, ACD, CCT (thinnest) and W–W.

### Surgical procedure

To proceed with the ICL implantation technique, two peripheral iridotomies were performed at least 1 week before the surgery using a neodymium:yttrium–aluminum–garnet laser in every patient. Preoperatively, we marked the zero and 180º horizontal corneal axis (TICL cases) while the patient was sitting upright to avoid potential cyclotorsion while lying supine. ICLs were sized according to corneal W–W distance and ACD measurements obtained by Orbscan II. The lens models used were TICM 115V4, 120V4, 125 V4, 130V4 and ICH 115V3, ICM 110V4, 115V4, 120V4, 125V4 and 130V4. All surgeries were performed under topical anesthesia; we marked the desired axis in TICL cases with a Mendez degree gauge (Katena Inc., Denville, NJ, USA). Two superior and inferior paracentesis incisions were performed, and a cohesive viscosurgical device was injected into the anterior chamber. A temporal clear cornea 2.8 mm incision was then made to inject the ICL or TICL. After the insertion of the lens, we placed the four haptics under the iris with a Batlle ICL manipulator (Asico LLC, Westmont, IL, USA) and aspirated viscosurgical devices; we used intraocular acetylcholine to achieve miosis. The correct positioning of the ICL in the center of the pupillary zone was verified as well as the patency of the iridotomies. We routinely preferred to use a single 10-0 nylon suture to close the main wound. In general we used a 7-day course of ciprofloxacin/dexamethasone ophthalmic suspension and oral acetazolamide 250 mg tablets twice a day over 3 days. Patients were examined at day 1, week 1, month 1, and every 3 months for a year, after which the follow-up was on a yearly basis with a complete ophthalmologic evaluation.

Subjects were evaluated according to the postoperative schedule. We evaluated age, preoperative UDVA and CDVA, objective and subjective refraction of both eyes, spherical equivalent, total keratometric power, and total astigmatism.

### Statistical analysis

Statistical analysis was performed using the Stata 8.0 software (StataCorp LP, College Station, TX, USA) using descriptive statistics (mean, standard deviation, rank). Paired *t* test was performed to identify differences in the preoperative and postoperative period, with *p* < 0.05 considered as significant.

## Results

In this study we describe visual and refractive outcomes of 216 consecutive patients (349 eyes) with toric and spheric Visian ICL phakic lens implantations for the management of refractive errors (myopia, hyperopia, myopic astigmatism and hyperopic astigmatism). Of the study patients, 66.2 % were female (143/216) and 33.8 % were male (73/216). Of the 349 eyes, 155 (44.4 %) eyes were treated with spheric ICLs and 194/349 (55.6 %) were treated with toric ICLs. 337 eyes had a diagnosis of some degree of astigmatism. The refractive error was myopic astigmatism in 328 eyes (94 %), hyperopic astigmatism in nine eyes (2.5 %), and isolated myopia in 12 eyes (3.4 %). Mean follow-up was 47 ± 31 months (3–127 months).

**Fig. 1:**
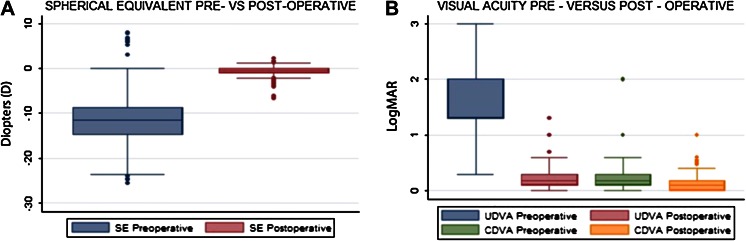
**a** Preoperative and postoperative spherical equivalent in patients treated with ICL implantation. **b** Pre- and post-operative UDVA (*p* < 0.001) and CDVA (*p* < 0.001) in patients treated with toric and spheric ICL implantation

The mean age of the patients was 29 ± 6.7 years (18–51 years). The mean preoperative sphere was −10.35 ± 5.1 D (+8 to −24) and the postoperative sphere was −0.09 ± 1.06 D (+3.25 to −6.5) (in one case the myopia was −24 D and the highest ICL power did not correct that amount of myopia) with a *p* value of <0.001. The preoperative cylinder was −2.63 ± 1.44 (0 to −6.5 D) and the postoperative cylinder was −0.97 ± 0.89 D (0 to −3.5) with a *p* value of <0.001. The preoperative mean spherical equivalent was −11.6 ± 5.12 D (+7.875 to −25.625) and the postoperative spherical equivalent was −0.52 ± 1.03 (+2.25 to −6.75) with a *p* value of <0.001 (Fig. 1a; Table 1).

**Table 1:** Pre- and post-operative clinical data comparison in patients who underwent ICL implantation

Measurement	Preoperative	Postoperative	*p* Value
Sphere	−10.35 ± 5.1 D	−0.09 ± 1.06	<0.001
Cylinder	−2.63 ± 1.44	−0.97 ± 0.89	<0.001
SphEq	−11.6 ± 5.12	−0.528 ± 1.03	<0.001
UDVA	1.72 ± 0.49	0.23 ± 0.22	<0.001
	(20/1,000)	(20/30)	
CDVA	0.21 ± 0.17	0.12 ± 0.13	<0.001
	(20/30)	(20/25)	

*SphEq* spherical equivalent, *UDVA* uncorrected distance visual acuity, *CDVA* corrected distance visual acuity

The mean preoperative UDVA was 1.72 ± 0.49 (mean 20/1050 Snellen, 0.3–3 logMar) and the postoperative UDVA was 0.23 ± 0.22 (mean 20/34 Snellen, 0–1 logMar) with a *p* value of <0.001. The mean preoperative CDVA was 0.21 ± 0.17 (mean 20/32 Snellen, 0–1 logMar) and postoperative CDVA was 0.12 ± 0.138 (mean 20/26 Snellen, 0–1 logMar (a patient with myopic choroidal neovascularization scar) with a *p* value of <0.001 (Fig. 1b; Table 1).

The mean keratometric values were 44.15 ± 1.968 (36.95–54.4), and mean ACD was 3.19 ± 0.28 mm (2.8–4.56). Mean corneal astigmatism was 2.447 ± 1.08 (0.02–5.6 D). The mean lCL vaulting value was 481 ± 185 μm (100–1,090).

**Fig. 2:**
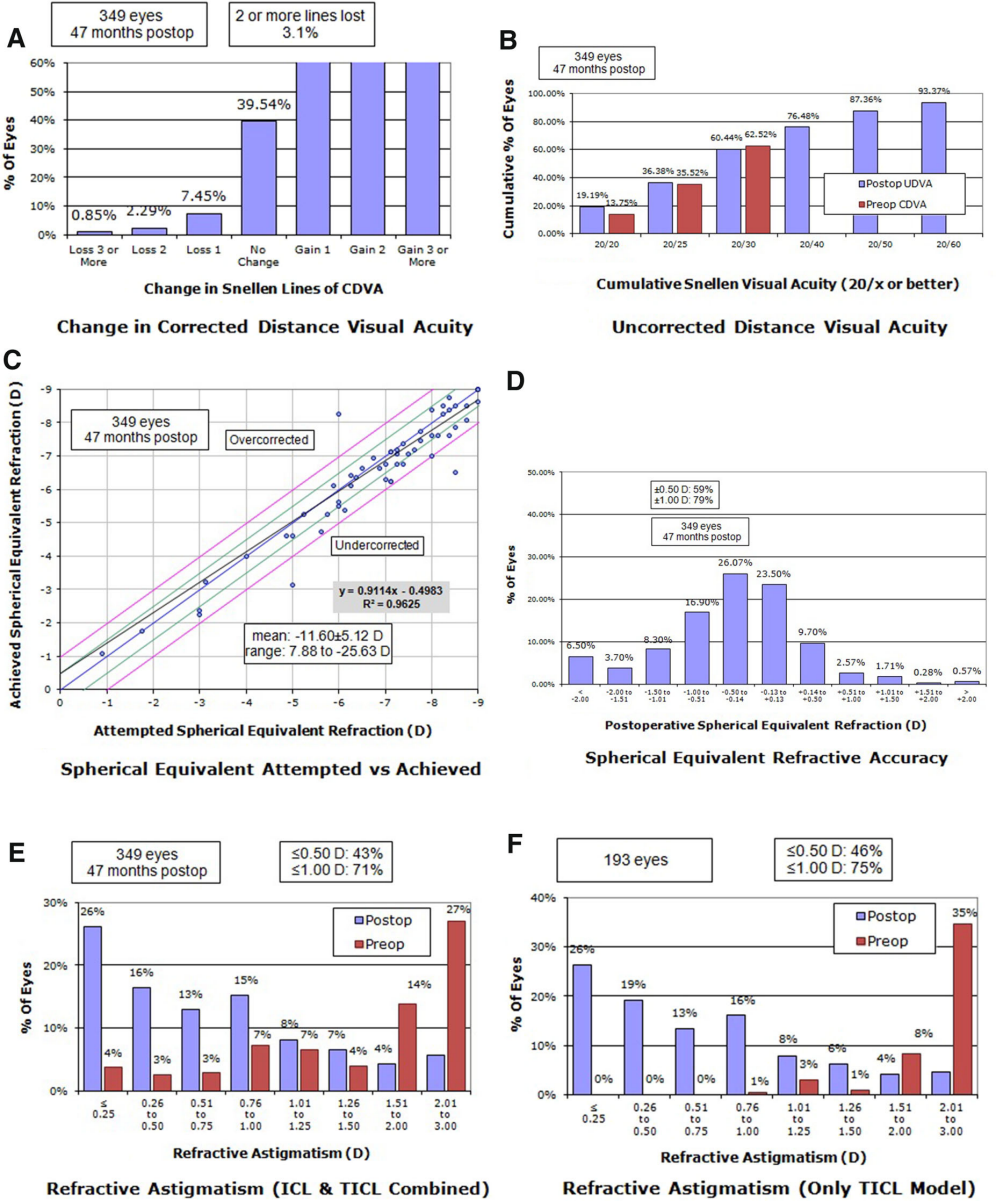
**a** Safety of the procedure. After the procedure, 92.55 % of patients had no change or gain in CDVA with the procedure. **b** Efficacy of the procedure. 76.5 % of patients achieved a postoperative UDVA of ≥20/40. **c** Predictability of the procedure with 98.10 % of correlation (*R* = 0.9625) between the attempted and the achieved spherical equivalent refraction. **d** Refractive accuracy of the procedure, with 59.27 % of patients within ± 0.5 D of residual error and 78.7 % within ± 1D of residual error. **e** Pre- and post-operative astigmatism (*p* < 0.001) in patients treated with toric and spheric ICL. **f** Astigmatism in patients treated with a TICL. The mean astigmatism correction with this IOL model was 74.9 %

The safety index of the procedure was 1.2, with 92.55 % of patients with no loss or gain of >1 line of CDVA after the procedure and only 2.29 % of patients with ≥1 lines lost (Fig. 2a). The effectiveness was 1.93 (Fig. 2b) and the predictability index was 21.96 with 98.10 % (*R* ^2^ = 0.9625) of correlation between the attempted and the achieved spherical equivalent with the procedure (Fig. 2c). The refractive accuracy of the procedure was high, with 59.27 % of patients with a postoperative refraction within ±0.5 D and 78.7 % within ±1 D of postoperative refractive error (Fig. 2d). The percentage of astigmatism reduction in the whole population was 61.5 % (0–100 %), and in the TICL models was 74.9 % (Fig. 2e, f). The postoperative astigmatism in the whole population was within ±0.5 D in 42.67 % and within ±1 D in 71 % of patients.

**Fig. 3:**
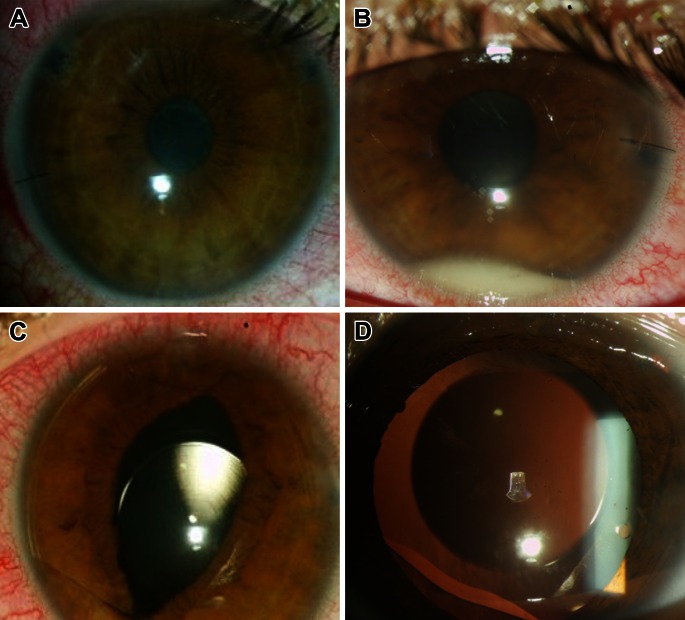
Lens and non-lens-related complications. **a** TASS syndrome, **b** hypopion in endophthalmitis, **c** TICL luxation after blunt ocular trauma and **d** focal anterior subcapsular cataract

The complication rate (Fig. 3) was 3.72 % (13 eyes), with 2 % of the total complication rate related to the lens and 1.72 % related to myopia or other ocular pathology (Table 2). We did not observe a significant amount of clinical crystalline lens opacity.

**Table 2:** Lens- (ICL) and non-lens (non-ICL)-related complications, management and visual outcome

No.	Preoperative UDVA/CDVA	Postoperative (final) UDVA/CDVA	Complication	Management
Lens-related complications				
1	2/0	0.176/0	Small vault	ICL exchange
2	1.3/0.176	0.3/0	ICL rotation	Surgical lens rotation (twice)
3	1.82/0.176	0.4/0.176	Toxic anterior segment syndrome	ICL removal
4	2.3/0.3	0.0969/0	Recurrent uveitis	Medical treatment
5	1.3/0.3	0.176/0.176	Recurrent ICL rotation	Surgical lens rotation (3 times)
6	2/0	0.176/0.176	Endophthalmitis	Intravitreal therapy and vitrectomy
7	1.18/0.4	0.176/0.0969	Postoperative open-angle glaucoma	Pharmacologic treatment
Non-lens-related complications				
8	2.3/0.54	0.7/0.477	Ocular trauma and macular hemorrhage with ICL luxation	Surgical lens reposition
9	1.7/0.0969	0.54/0.397	Myopic choroidal neovascularization	Antiangiogenic therapy
10	1.7/.3	0.176/0.0969	Previous retinal detachment	Post-scleral buckle ICL implantation
11	2/1	1/1	Retinal scar from previous myopic choroidal neovascularization	
12	1.3/0	0.3/0.3	Ocular trauma with secondary ocular hypertension and endothelial failure	DSAEK
13	1.3/0.0969	0.54/0.54	Post-ICL rhegmatogenous retinal detachment with macular involvment	Scleral buckle

*DSAEK* Decemet’s stripping automated endothelial keratoplasty

## Discussion

Our results confirm the potential benefits of phakic collamer lens implantation as an effective treatment option in the refractive surgery armamentarium; we found a remarkable improvement in visual acuity and refraction with both clinical and statistical significance.

As with any intraocular surgery, ICL complications can occur and they can be devastating [13]. We presented one case of toxic anterior segment syndrome (TASS, Fig. 3a), in which we decided to explant the ICL. It was also a case of culture-positive *Staphylococcus epidermidis* endophthalmitis (Fig. 3b) that we were able to treat opportunely, remaining with an adequate UDVA of 20/40. Few previous studies have reported endophthalmitis after ICL, showing an estimate rate of 0.0016 (0–0.036 %) [13, 14].

Overall, the findings of this study showed that pIOLs can be safe with an adequate safety index of 1.2. It is reasonable to be concerned about potential complications [15], but large long-term series are very helpful to demystify the bad reputation of phakic IOLs that could be considered an excellent and adequate alternative in well-selected cases.

Some studies, including FDA trials have shown both the safety and efficacy of pIOLs [9, 12, 16]. Our study has an adequate follow-up of 47 months allowing us to evaluate long-term complications. While it seems that our results had a high complication rate, fortunately most of them had a good final outcome. A key factor is ICL sizing; we used Orbscan II for all biometric measurements, but these criteria may vary among different populations [17]. We consider it extremely important to be strict in patient selection and to respect the measurement limits. The achieved versus the attempted refraction showed an excellent correlation of 98 % (*R* = 0.9810), which was clinically reflected by noticeable subjective patient satisfaction.

While our mean preoperative spherical equivalent was approximately −11 D, it is difficult to establish a refraction cut-off to implant pIOLs. Recently Hardten [18] published an editorial article arguing that pIOLs should not be considered just in extreme and rare cases. Gradually pIOLs are gaining more indications. There are few studies that compare visual quality; however, those that do, have concluded that it can be similar or even better than LVC including laser in situ keratomileusis, photorefractive keratectomy and wavefront-guided ablations [19–21].

This study in combination with other large series [9, 12] could give us a different perspective of posterior chamber pIOLs and we should consider them an alternative in refractive treatment. Regarding toricity, we considered that in cases of <2 D of astigmatism the spheric version can be sufficient to resolve the refractive error, while in cases of >2 D of astigmatism the TICL model is preferable, and we used as needed when TICL became available. We presented just one case of TICL spontaneous rotation [22], and a few intraoperative misalignments, all of them solved by repositioning the TICL.

Nowadays, pIOLs are considered in patients with contraindication for other refractive surgery procedures. The design and materials of this type of lens are improving notably over time [23, 24]. LVC is an excellent procedure that is well performed and studied around the world [25], but the refractive range of LVC is becoming less wider than in the past. Perhaps, in the near future more surgeons will also consider pIOLs in regular cases and not just in cases where LVC could involve risks [18].

Advantages of pIOLs include a large range of refractive errors, and the fact that the necessary surgeon’s skills are similar to cataract surgery. It is a reversible or removable procedure, preserves the natural accommodation, and has a lower risk of retinal detachment compared with refractive lens exchange [26]. Disadvantages include the potential risk of intraocular surgery including endophthalmitis, development of iatrogenic cataracts, pupillary block and endothelial loss [15].

The decision between choosing an anterior chamber versus posterior chamber pIOL is very debatable. The most important argument against anterior chamber pIOLs is the potential endothelial damage [1, 2] versus the main criticism of posterior chamber pIOLs which is cataract formation [4, 5]. In our retrospective review few focal anterior subcapsular cataracts formed (Fig. 3) but none was visually significant to require pIOL removal. In the scenario that cataract surgery is needed, the IOL calculation does not seem to be an important issue [27], because there is a no significant difference in axial length and keratometries after ICL implantation, and phacoemulsification after ICL has been previously reported without important considerations [28]. On the other hand, we presented a case (Table 2) that underwent endothelial keratoplasty due to ICL dislocation after trauma and subsequent endothelial decompensation [29].

Our study has several limitations, one of them being that different surgeons (including surgeons under training) performed the procedures as this can change or modify the complications rate. Another limiting factor is the measurement of endothelial cell loss because not all the cases completed measurements or long-term follow-up. Furthermore, our specular microscopy was upgraded; hence, different software counts between preoperative and postoperative measurements would not allow an adequate statistical analysis. Nevertheless, the data obtained were 2,409 cells/mm^2^ (SD 443.88) preoperative versus 2,385 cells/mm^2^ (SD 410.98) postoperative with a *p* value of 0.3636 (paired *t* test) over an average of 75 months (36–110) follow-up. Another limitation was that most patients had the same ethnicity where ICL sizing was very acceptable, as this may vary in different countries and populations [17], while ultrabiomicroscopic measurements could provide the most accurate sulcus-to-sulcus distance [30]. We used Orbscan II consistently for all biometric values.

Newer improvements are developing in pIOLs. In particular, the toric marks of the ICL are closer to the pupil to help achieve a better intraoperative alignment without needing pupil dilatation to evaluate the toricity alignment during follow-up. Furthermore, newer pIOL models [10, 24] promise that no iridotomies would be needed and lenses could be available in pre-loaded cartridges. Further research studies are necessary in the future with regard to materials, lens design [11, 23], rotation and intraocular flow dranaige. In conclusion, while the most feared complications can occur after ICL implantation, they can also be solved satisfactorily. ICL implantation is a safe and effective procedure in refractive surgery.
